# Patch Testing with Disperse Blue Mix and Textile Dye Mix in Textile Dermatitis: Diagnostic Effectiveness, Co-Positivity and Clinical Relevance

**DOI:** 10.3390/jcm15082936

**Published:** 2026-04-12

**Authors:** Radoslaw Spiewak

**Affiliations:** Institute of Dermatology, ul. Kustronia 2, 30-433 Krakow, Poland; r.spiewak@instytutdermatologii.pl

**Keywords:** textile dermatitis, textile dye allergy, screening, patch testing, diagnostic haptens, hapten mixes

## Abstract

**Background/Objectives**: Textile dermatitis seems underdiagnosed, partly due to low awareness of this problem and partly due to imperfect screening methods. The aim of this study was to analyse the diagnostic efficacy and clinical relevance of two textile dye mixes used in routine patch testing. **Methods**: Retrospective analysis of patch test results and clinical records of patients tested with textile dyes in a specialised patch test practice. **Results**: Between 2007 and 2024, 207 patients were patch tested with Disperse Blue Mix 106/124 (DBM). Positive reactions were observed in 17.4% of patients, including 10.6% considered clinically relevant. Between 2019 and 2023, 90 patients were tested with Textile Dye Mix (TDM) 6.6%, of whom 14.4% developed a positive reaction, relevant in 4.4%. In a subgroup tested with TDM 6.6%, DBM, all their components and cross-reacting azo dyes, out of eight patients with confirmed textile dermatitis, three cases would have been missed if screening had been performed using TDM alone (6.6%), compared with one case being missed if screening had been performed using DBM alone. The highest rate of positivity (78.6%) to TDM 6.6% was found among patients with an allergy to the common hair dye Toluene 2,5–Diamine Sulfate, all of whom were also positive to Disperse Orange 3 (DO3) present in TDM 6.6%. Among patients with positive tests to Disperse Blue (DB) 106 1% pet. or DB124 1% pet., 68.7% and 85.7%, respectively, reacted also to DBM 106/124 (each component at 0.5%), with respective figures for TDM 6.6% (DB106 and DB124 each at 0.3%) amounting to a mere 12.5% and 14.3%. **Conclusions**: The detection rates of textile dermatitis can be increased by improving screening tools for textile dye allergy in baseline series for routine patch testing. Based on the results of this study, proposed improvements include removing Disperse Orange 3 from the textile dye mix, tripling the concentrations of Disperse Blue 106 and Disperse Blue 124 in the textile dye mix, and doubling their concentrations in the Disperse Blue Mix 106/124.

## 1. Introduction

Textile dermatitis is difficult to diagnose because of the variable morphology and distribution of eczema and the lack of telltale symptoms in most cases. Therefore, cases of textile dermatitis are likely to be missed or misdiagnosed, e.g., as atopic dermatitis [1,2,3]. In more than 20% of patients with dermatitis, contact allergy to textile dyes may fully or partially explain their disease [4]. The growing awareness of the importance of clothes as an important source of sensitisers has led to the inclusion of selected textile dyes into international and national baseline series for patch testing, mainly Disperse Blue 106 or Disperse Blue Mix 106/124 (DBM). The inclusion of the Textile Dye Mix 6.6% pet. (TDM 6.6%) into the European Baseline Series (EBS) was proposed in 2015 and formally accepted in 2019 [5,6]. Also in 2019, TDM 6.6% was included in the Polish Baseline Series listed in the manual of good clinical practice in allergy of the Polish Society of Allergology [7]. The original TDM 6.6% was a mix of eight textile dyes in petrolatum at the following concentrations: Disperse Blue 35 (DB35) 1%, Disperse Yellow 3 (DY3) 1%, Disperse Orange 1 (DO1) 1%, Disperse Orange 3 (DO3) 1%, Disperse Red 1 (DR1) 1%, Disperse Red 17 (DR17) 1%, Disperse Blue 106 (DB106) 0.3%, and Disperse Blue 124 (DB124) 0.3%; thus, the cumulative dye concentration was 6.6% [6]. However, from the very beginning, this composition became a subject of controversy, as summarised in [8]. Especially, the presence of DO3 in the mix had been contested because of its cross-reactivity with common hair dyes [9].

The author has been patch testing with textile dyes since 2007, initially only in patients with suspected textile dermatitis. Starting from 2012, all patients qualified for routine patch testing in our clinic were tested with an in-house, extended series that included Disperse Blue Mix 106/124 (DBM) 1% pet., consisting of DB106 and DB124, each at 0.5%. Bearing in mind the above controversies, during an overhaul of the in-house series in 2019, the newly recommended TDM 6.6% was added to DBM instead of replacing it. Additionally, the series was supplemented with DO3 in order to single out patients with possible cross-reactions to non-textile azo dyes. Considerations about insufficient concentrations of DB106 and DB124 in DBM (0.5% each) and especially in TDM 6.6% (0.3% each) inspired the addition of these two dyes as separate test substances, each at 1%. In the end, every patient qualified for routine patch testing had five substances placed on their back with the sole purpose of a reliable screening for textile dye allergies. Although this seemed not sustainable in a long-term perspective, it also was considered the only way of ensuring an efficient and bias-free screening available at that time. This retrospective analysis of patch test results with textile dyes was undertaken in order to compare the diagnostic efficacy of available screening mixes for textile dye allergies in the context of their clinical relevance.

## 2. Materials and Methods

This retrospective analysis included all patients tested in our clinic with Disperse Blue Mix 106/124 1% pet. (DBM, Chemotechnique Diagnostics AB, Vellinge, Sweden; Art. No. Mx–26) starting from April 2007 until the end of the observation period in March 2024. Initially, these results were acquired during a diagnostic workup of patients with a suspicion of textile dermatitis. Since 2012, all patients qualified for patch testing, except special cases like implant or dental allergy, were tested with an in-house series that included DBM, and from 2019, also DB106 (D–040) and DB124 (D–041). From October 2019, all patients were also routinely patch tested with Textile Dye Mix 6.6% pet. (TDM 6.6%, Mx–30) until May 2023, when the last batch of the mix expired and a replacement was no longer available. Among patients tested with TDM 6.6%, special attention was paid to those who were tested in parallel with all its individual ingredients: DB35 1% pet. (D–027), DO1 1% pet. (D–031), DO3 1% pet. (D–032), DR1 1% pet. (D–034), DR17 1% pet. (D–035), DY3 1% pet. (D–036), DB106 1% pet. (D–040), and DB124 1% pet. (D–041). Another subgroup of special interest included patients tested with TDM 6.6% and, in parallel, with DO3 1% pet. (D–032) and other dyes cross-reacting with DO3, i.e., para–Phenylenediamine 1% pet. (PPD, P–006), Toluene Diamine Sulfate 1% pet. (TDS, D–002), and 4–Aminoazobenzene 0.25% pet. (4AAB, A–005). The haptens were applied on the patient’s back or thighs in IQ Chambers, IQ Ultra, or IQ Ultimate test units. All diagnostic materials presented in this article were from Chemotechnique MB Diagnostics AB (Vellinge, Sweden). Whenever it seemed justified and feasible, the patients were also tested with patches of their own suspected garments.

All patch tests were personally applied and assessed by the author, a dermatologist and allergist who at the beginning of the observation period, had practical experience in patch testing of more than 15 years. Throughout the entire observation period of 17 years, an in-house patch testing protocol was followed that was consistent with the ESCD guidelines of 2015, including 2 days of occlusion and a minimum of 3 readings within the subsequent 5 days [10]. The only deviation from the ESCD guidelines was the numbering of days for subsequent readings, which in Poland are traditionally counted from day 1 (D1, starting day), rather than from day 0. Such numbering seems more intuitive and is also used in other countries, e.g., by the Mayo Clinic Contact Dermatitis Group [11]. The consecutive readings in all patients were done on D3 (D2 according to ESCD), D5 (D4) and D8 (D7). In line with the ESCD guidelines, an overall patch test result was considered positive if there was at least one reaction fulfilling the ICDRG criteria of a positive reaction on any of the reading days. Clinical relevance was assessed with the help of the CODEX grading system used throughout the analysed period [12]. Patch test results were extracted from patients’ records and coded for further analyses. Records and clinical photographs of all patients with positive reactions were critically analysed in order to verify the diagnosis of textile dermatitis. A positive patch test reaction to a textile dye was considered clinically relevant when the eczema was limited to or visibly more pronounced in skin areas of direct contact with clothing made of a fabric and colour consistent with the dye, e.g., dark blue synthetic clothing in the case of positive patch test results with disperse blue dyes. Further findings supporting the clinical relevance were a positive reaction to fragments of the suspected clothing or a reduction in the perceived burden of dermatitis by at least 50% after discontinuation of wearing it. Positive patch tests were interpreted as cross-reactions in patients with co-existing positive patch tests to haptens known to cross-react with these textile dyes (e.g., hair dyes) and the clinical picture indicating the cross-reacting haptens as the primary cause of dermatitis (e.g., relapses of facial dermatitis after hair dyeing). The decision about clinical relevance was partly circumstantial due to the lack of information about the actual dye content in the offending clothes. Therefore, in order to enable the readers to make their own opinion, the key characteristics of the patients with patch tests assessed as relevant, as well as those with cross-reactions, are shortly presented.

While analysing the patch test results and clinical data, the following aspects were taken into consideration:

- The rates of positive patch test reactions to DBM and their clinical relevance, as well as patterns of co-positivity in patients who were tested with both DBM and its components DB106 and DB124;
- The rates of positive patch test reactions with TDM 6.6% and their clinical relevance, as well as patterns of co-positivity in patients who were tested with both TDM 6.6% and all its components DB35, DY3, DO1, DO3, DR1, DR17, DB106, and DB124;
- The patterns of co-positivity and clinical relevance in patients tested with TDM 6.6% and in parallel with DO3, as well as with cross-reactive azo dyes PPD, TDS and 4AAB.

## 3. Results

### 3.1. Reactions to Disperse Blue Mix 106/124 and Its Components

Altogether 207 patients, 148 females and 59 males aged 6–75, median 32 years, were tested with DBM during the observation period. Doubtful reactions (“?”) were recorded in 66 (31.9%, 95%CI: 25.5–38.2%) patients and weak positive reactions in 36 (“+”, 17.4%, 95%CI: 12.2–22.6%); strong reactions were not observed. Positive patch test reactions were deemed clinically relevant in 22 patients (10.6%, 95%CI: 6.4–14.8%). Forty-three patients were tested with both DBM and its ingredients: 22 females and 21 males aged 6–63, median 29 years. In this group, a positive patch test reaction to at least one of these haptens was found in 19 (44.2%, 95%CI: 29.3–59.0%) patients, including 13 (30.2%, 95%CI: 16.5–44.0%) in whom these reactions were assessed as clinically relevant. Co-positivity between DBM and its components is shown in Table 1.

**Figure 1:**
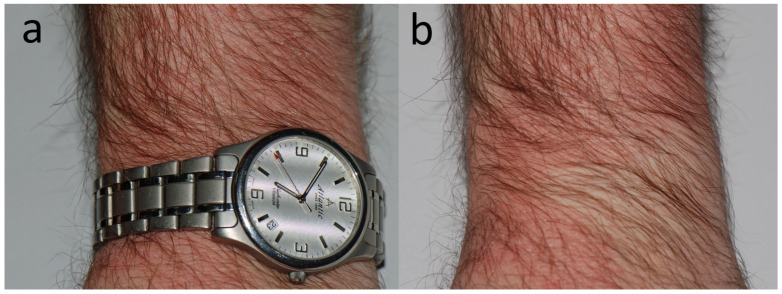
Patient A, a Catholic priest (M, 46 y.o.) who complained of recurrent, large, irregular and poorly demarcated patches of mild to moderate dermatitis on the trunk and upper limbs, accompanied by mild pruritus that lasted over a year. Skin under his wrist watch (**a**) was spared (**b**) which directed the suspicion toward his black priestly shirt. The eczema has subsided after he started wearing a long-sleeved white cotton underwear under the black shirt.

**Figure 2:**
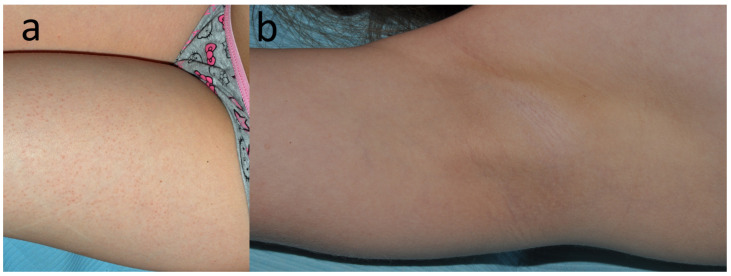
Patient B, a schoolgirl (8 y.o.) with chronic, recurrent pruritic eczema since 3 y.o. The eczema on her thighs showed a follicular pattern (**a**); treatments for keratosis pilaris were ineffective. Hyperpigmented dermatitis in the groins, popliteal fossae, ankles and dorsal feet. The involvement of axillary folds with sparing of vaults (**b**) directed the suspicion toward textile dermatitis. According to the patient’s mother, avoidance of dark synthetic textiles resulted in a “great improvement”.

**Figure 3:**
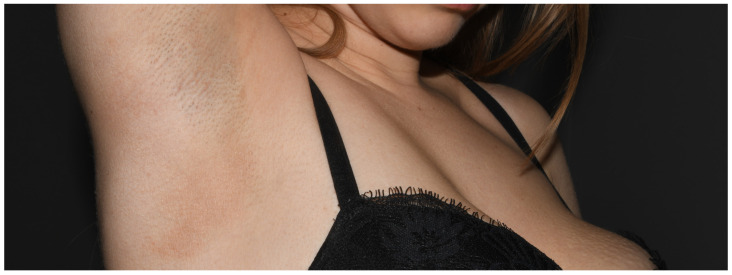
Patient C, a waitress (27 y.o.) with recurrent pruritus and dermatitis since 12 y.o. Eczematous patches are more pronounced in the elbow fossae, arms, trunk, buttocks and thighs. Involvement of axillary folds with sparing of armpits was suggestive of textile dermatitis. Other than DBM, DB106 and DB124, patch tests were also positive to Disperse Brown 1 and Reactive Black 5—all were deemed clinically relevant based on improvement in skin condition after phasing out black, blue, and red clothing.

**Figure 4:**
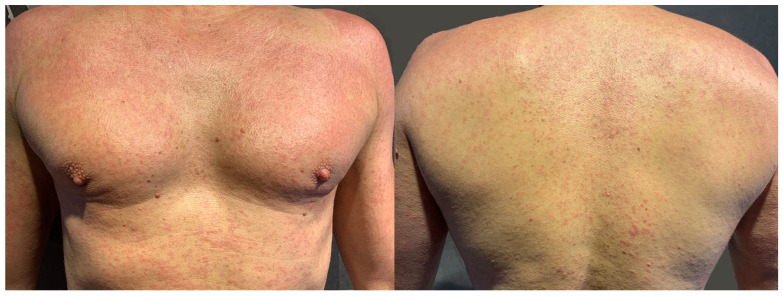
Patient D, a shopkeeper (M, 53 y.o.) with itchy, dispersed dermatitis that persisted over 2 years. In the shoulder and pectoral regions, eczema is most pronounced in areas of tight contact with his T-shirts, which he preferred in dark colours.

**Figure 5:**
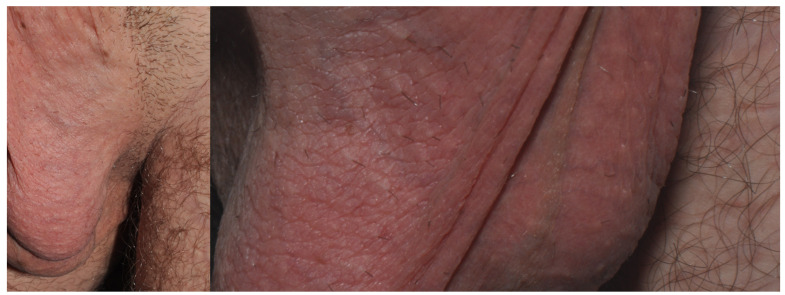
Patient E, a dentistry student (M, 25 y.o.) with recurrent itchy lichenified dermatitis of the scrotum and penis that had lasted for 7 years. He usually wore black or navy blue underwear. A change to white underwear resulted in a significant improvement.

**Figure 6:**
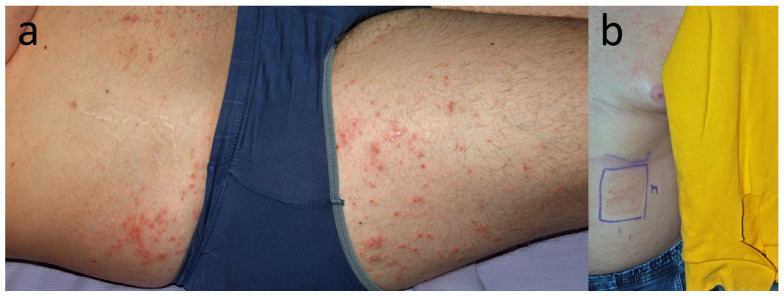
Patient F, a middle school student (M, 15 y.o.) with recurring eczema that lasted over 3 months. (**a**) Dispersed dermatitis is most pronounced around the waist and under slips (mostly dark colours). Patch test positive to DBM, DB106, and DB124. (**b**) Eczematous reaction to a patch from his own yellow hoodie, pictured on D5, i.e., 2 days after removal of the patch.

**Figure 7:**
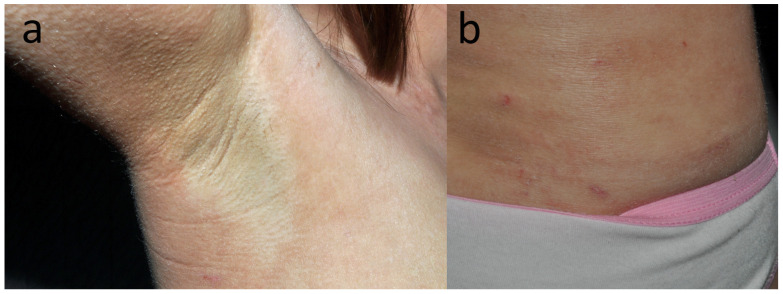
Patient G, a dancer (F, 27 y.o.) with chronic, dispersed dermatitis that lasted over 4 years, starting from the popliteal fossae and spreading over the legs, trunk and neck. The distribution of dermatitis in the axillae (**a**) and accentuation of eczema under the elastic band of her panties (**b**) were suggestive of textile dermatitis. A change of clothes from black to white resulted in a 50% improvement.

**Figure 8:**
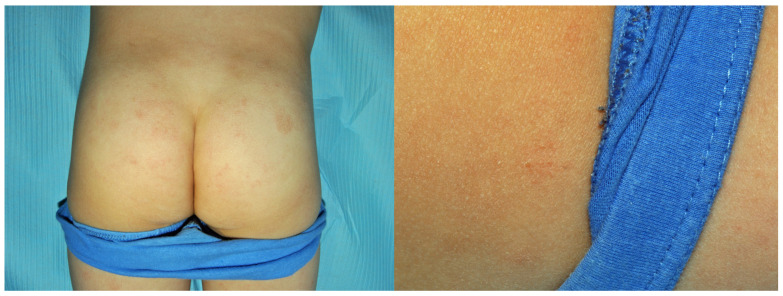
Patient H, a boy (6 y.o.) presented with dispersed patches of dermatitis on the shoulders and upper and lower limbs. Recurrent eczema since 1 y.o., more pronounced and lichenified under blue slips, which was the typical colour worn. Other than DBM and DB124, a patch test was also positive to DB35.

**Figure 9:**
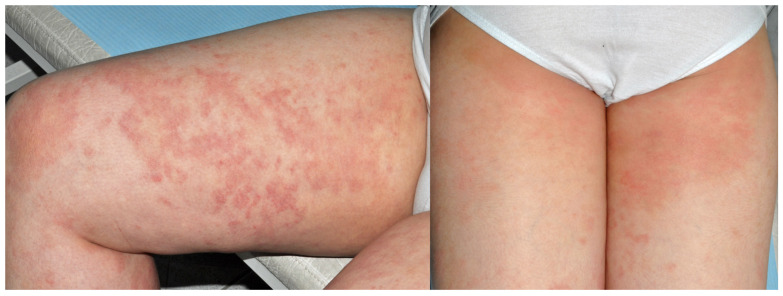
Patient I, a schoolboy (12 y.o.), according to parents “born with skin problems”. He presented with patches of eczema, most pronounced on the thighs. The skin under white panties was spared; therefore, his blue jeans and trainers were suspected. Patch tests were also positive to DB85.

**Figure 10:**
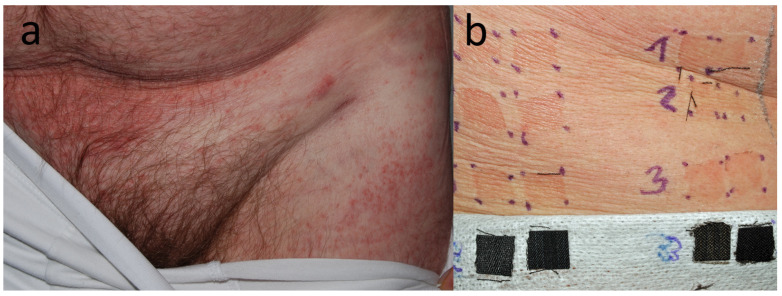
Patient J, a salesman of men’s clothing (50 y.o.). For 5 months, eczematous patches were spreading over the entire body. (**a**) The skin under his briefs seemed spared on the hips and buttocks but not on the pubic mound; later, PT revealed nickel allergy, hinting at belt buckles as the cause of dermatitis in this particular location. (**b**) Positive reactions to clippings of his own and traded suits, along with positive patch test reactions to textile dyes confirmed the diagnosis of textile dermatitis.

**Figure 11:**
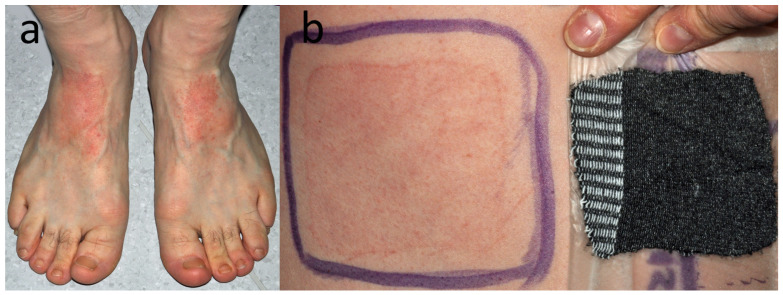
Patient K, a carpenter and flooring installer (M, 39 y.o.) with recurrent hand and (**a**) dorsal foot eczema since 20 y.o. (**b**) Positive reaction to a patch from his black sock, here pictured on D5.

**Figure 12:**
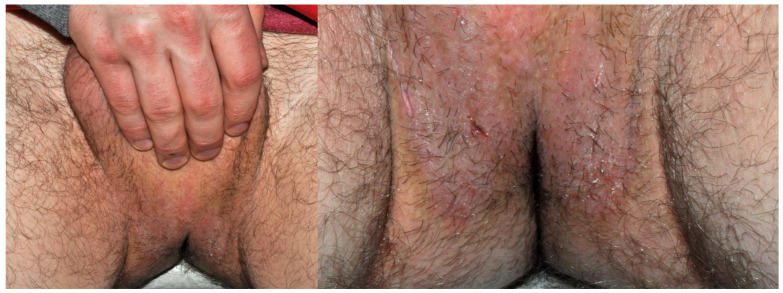
Patient L, a seamster and ironer (M, 28 y.o.) with chronic dermatitis on the lower aspects of the scrotum and perineum that had lasted for 2 years, recently also had hand eczema. Aside from DB106, he was also positive to DO34 and 4AAB, but not to DO3.

**Figure 13:**
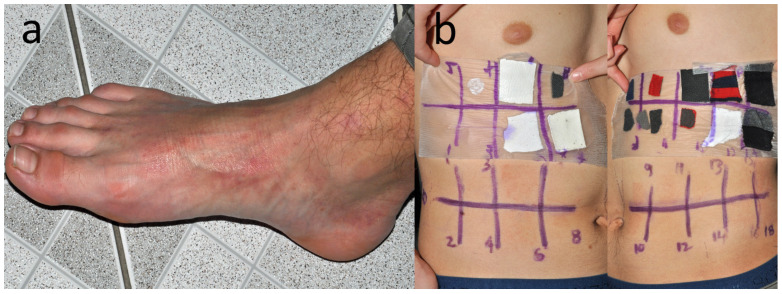
Patient M, a middle school student (M, 15 y.o.) with eczema on dorsal feet (**a**) which appeared 2 years earlier when he was taking swimming lessons and often came out of the pool with wet socks. Patch tests are also positive to Reactive Red 238. (**b**) Eczematous reaction to samples of own socks and shoe linings on D5.

As shown in Table 1, the majority of DBM-positive patients reacted to at least one of its ingredients. However, two patients in the last column reacted only to DBM but not to its components; in both, the final diagnosis was other than textile dermatitis. The key features of remaining patients whose history, combined with positive patch test results, led to the ultimate diagnosis of textile dermatitis are presented in Figure 1, Figure 2, Figure 3, Figure 4, Figure 5, Figure 6, Figure 7, Figure 8, Figure 9, Figure 10, Figure 11, Figure 12 and Figure 13. Please note that some of these patients may have had concomitant diagnoses with other significant patch test reactions which have been omitted here if deemed irrelevant to the main topic.

### 3.2. Patients with Clinically Relevant Patch Test Reactions to Textile Dye Mix 6.6%

**Figure 14:**
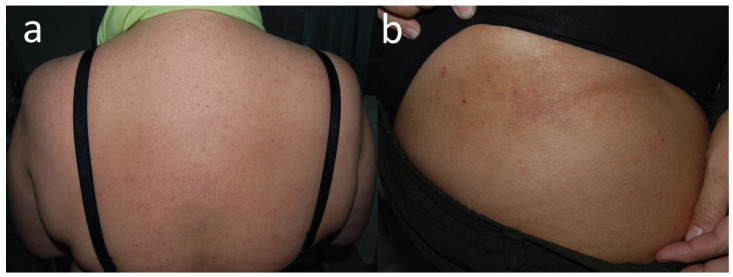
Patient N, a translator (F, 25 y.o.) with eczema dispersed over areas covered by clothing (**a**) that emerged a few weeks before. (**b**) Eczema was more pronounced under her black panties.

**Figure 15:**
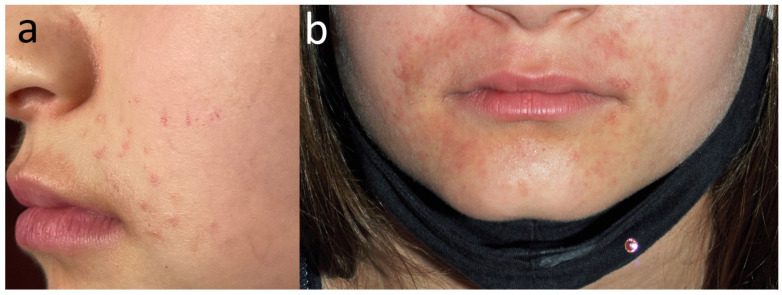
Patient O, a school student (F, 11 y.o.), during the COVID-19 pandemic sought help for her cheilitis that had lasted then for 3 years. (**a**) On the first visit, inflammatory papules were also noted around her mouth, initially thought to be acne papules; however, they subsequently coalesced into patches of eczema confined to the lower half of the face. (**b**) Only the positive patch tests to TDM 6.6% and DBM made the patient realise that the problems on her cheeks appeared shortly after the introduction of the obligation to wear a face mask, and she opted for a textile navy blue mask. Her cheilitis was due to linalool (positive patch test) in her toothpaste (positive semi-open tests).

**Figure 16:**
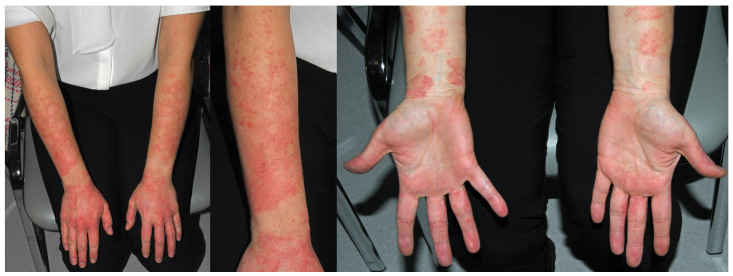
Patient P, a surgeon (F, 30 y.o.) developed dermatitis on the forearms in the preceding year, involving only areas in direct contact with the sleeves of her dark blue surgical gowns. Upper arms were protected by short sleeves of her undershirt and wrists by elastic sleeve cuffs. Concomitant hand dermatitis was due to neoprene surgical gloves, as well as surgical cleansing emulsion from the operating theatre.

In the period from October 2019 to May 2023, 90 patients (67 women and 23 men aged 6–75 years) were patch tested with TDM 6.6%, which at that time was both in the baseline series and the textile series. Among them, 40 developed a doubtful (“?”, 44.4%, 95%CI: 34.2–54.7%), 10 a weak positive (“+”, 11.1%, 95%CI: 4.6–17.6%), two a strong (“++”, 2.2%, 95%CI: 0.0–5.3%), and one an extreme (“+++”, 1.1%, 95%CI: 0.0–3.3%) reaction. Out of the 13 (14.4%, 95%CI: 7.2–21.7%) patients with positive reactions, clinical relevance was found in four (4.4%, 95%CI: 0.2–8.7%), of whom patient D was presented above. Clues leading to the current relevance of positive patch test results and the diagnosis of textile dermatitis in the remaining three patients N, O, and P are presented in Figure 14, Figure 15 and Figure 16.

### 3.3. Patients Tested with Textile Dye Mix 6.6% and All Its Components

In the analysed period, 10 patients were tested with TDM 6.6% and all its eight components. They were also tested with three azo dyes cross-reactive with DO3 (Table 2).

**Figure 17:**
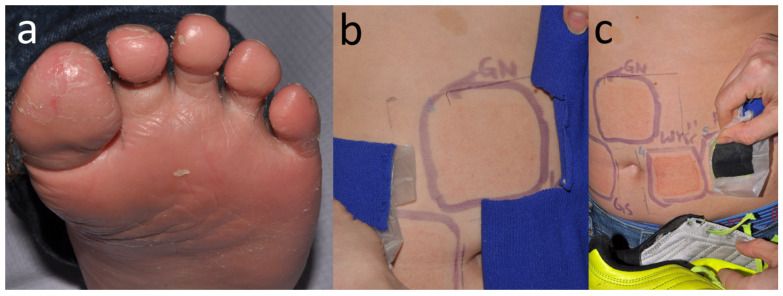
Patient S, a primary school student (M, 12 y.o.), complained of foot eczema that had lasted for 4 years (**a**). It reportedly started a couple of months after joining a football school. On patch testing, he developed a positive reaction to Acid Red 118 (not in the TDM 6.6%). Application of samples from his football kit revealed positive reactions to his socks (**b**), as well as the insole and inner lining of his football shoe (**c**).

As shown in Table 2, positive reactions to TDM 6.6% were observed in four patients. Two of them (cases D and G) developed weak positive reactions to TDM 6.6%, and in parallel, they also tested positive reactions to DB106, DB124 and DBM. As already presented, both were ultimately diagnosed with textile dermatitis. Further, patient F did not react to TDM 6.6% but developed positive reactions to its components DB106 and DB124, as well as DBM. He was finally diagnosed with textile dermatitis, which was confirmed by the eczematous reaction to a patch from his own hoodie, as shown in Figure 5. Another two patients developed strong or extreme reactions to TDM 6.6%; both were also positive to DO3 and cross-reactive azo dyes 4AAB, PPD and TDS. One of them, patient Q, was a male mining engineer (27 y.o.) who complained of hand eczema that started in his childhood. A year before patch testing, for the first time, he also developed foot eczema and dermatitis scattered over his body and extremities. His sensitisation to PPD and other azo dyes was linked to an episode of dermatitis after a temporary “henna” tattoo at 8 y.o., which reportedly caused a “scar” that persisted for more than 3 years. Relevant reactions were found to fragrances, his own cosmetics and fragments of four smartphone cases. The second patient, R, was a male hairdresser (34 y.o.), 13 years in the profession. Within 2 years preceding the referral, he developed hand eczema that aggravated after contact with hair dyes and other cosmetics in his hair salon. Eczema initially affected only the right (dominant) hand, later spreading also on the left hand. He reported remissions while on vacation. Next to positive reactions listed in Table 2, he also had positive reactions to 3–Aminophenol, 4–Aminophenol, Hydroxyethyl–P–Phenylenediamine Sulfate, Toluene–2,5–Diamine, Ammonium Persulfate, Benzyl Paraben, Hydroperoxides of Linalool, Colophonium, Lanolin, Amerchol L–101, Fragrance Mix I, Fragrance Mix II, propolis, Ylang–Ylang Oil, Gallate Mix, Formaldehyde, and Methylisothiazolinone. Semi-open tests were positive to five hair dye products from his hair salon. In both patients, Q and R, textile dermatitis was not confirmed, and their strong reactions to TDM 6.6% were probably due to cross-reactions between the non-textile azo dyes and OD3 in the mix. In the remaining five patients, doubtful or negative reactions to TDM 6.6% and its components were recorded. Interestingly, one of them (patient S) was ultimately diagnosed with textile dermatitis, as shown in Figure 17.

### 3.4. Textile Dye Mix 6.6%, Disperse Blue Mix 106/124, DO3 and Cross-Reactive Azo Dyes

In the analysed period, 85 patients were tested with TDM 6.6%, DBM, and DO3, as well as cross-reactive azo dyes PPD, TDS and 4AAB. At least one positive reaction to any of these haptens was recorded in 18 (21.2%) patients. Their results are shown in Table 3.

**Figure 18:**
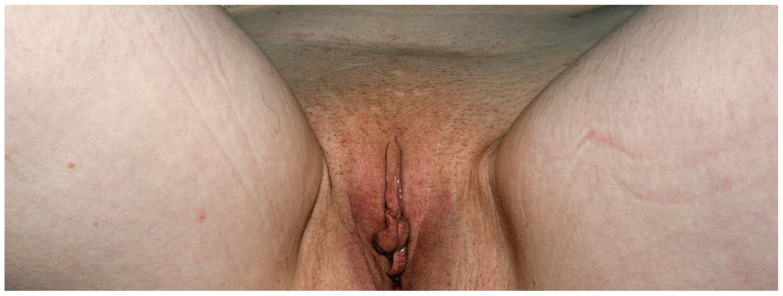
Patient T, an office clerk (F, 33 y.o.) who complained of recurrent perianal and vulvar dermatitis that had lasted for 5 years. After learning about the positive patch test to DBM, she stopped wearing dark-coloured underwear and noticed satisfactory improvement within a month.

**Figure 19:**
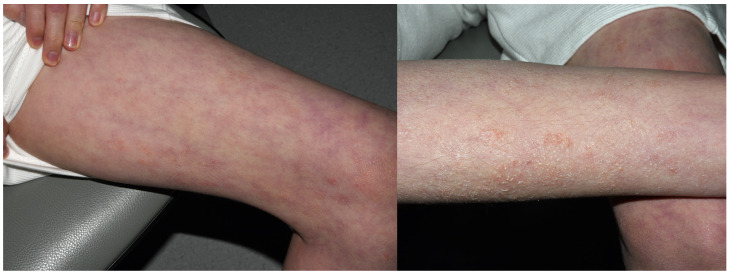
Patient U, a middle school student (M, 14 y.o.), complained of recurrent, symmetrical, diffuse, scaly eczema on the legs and in ulnar fossae persisting for more than a year. According to his mother, he had aggravations when wearing long pants, which she associated with sweating rather than fabric or colour. Other than DBM, patch tests were also positive to Fragrance Mix I, Myroxylon pereirae, and methylisothiazolinone. Semi-open tests revealed an eczematous reaction to his body-cleansing cream and emollient body emulsion. Five months later, the mother admitted on a follow-up visit that avoiding culprit cosmetics, as well as blue and dark textiles, resulted in 5 months’ remission, except for one episode of “red itchy spots” on the neck that appeared after wearing a new, navy blue scarf.

Patients presented in Table 3 can be divided into three distinct patch test patterns. The first pattern (P1) was represented by four patients who reacted only to DBM. Three of them (patients F, T, and U) were ultimately diagnosed with textile dermatitis, and their positive test results to DBM were considered clinically relevant. The justification for patient F has been presented earlier (Figure 6); the key clinical features of the remaining two patients, T and U, are presented in Figure 18 and Figure 19.

The second pattern (P2) was represented by six patients who reacted to both DBM and TDM 6.6%, but no other dyes listed in Table 3. Textile dermatitis was diagnosed in four of them (patients D, G, M and O), amounting to 66.7% positive reactions found relevant for both mixes. Further, seven patients in Table 3 presented another distinct pattern (P3) consisting of positive reactions to TDM 6.6%, DO3 and cross-reactive azo dyes but not to DBM. Next to the above-described patients Q and R, this subgroup also featured patients V, W, X, Y and Z. Patient V was a family doctor (F, 32 y.o.) who, 7 years earlier, had an episode of “dermatitis of the entire head” which started 2 days after hair dyeing and lasted for over a month. She also remembered two earlier episodes of localised dermatitis from temporary tattoos painted by beach artists. In this patient, semi-open tests were positive for 13 out of 17 various hair dyes that she brought for testing in the search for a product that would be safe for her to use. Further, two patients in this group seemed to have contracted an allergy to PPD from dyeing their eyebrows. This was the case of patient W (F, 36 y.o.), a saleswoman of sports articles, who complained of “atopic dermatitis that lasted for many years”. Her eczema was mainly located on the face, neck, flexures and hands. She reported that she had dyed her eyebrows with “black henna” 2–3 times a year for more than 15 years. A year before testing, such dyeing provoked an itchy dermatitis of the forehead and eyelids that had never fully resolved since then. Next to the azo dyes in Table 3, she also reacted to her own shampoos on semi-open tests. A similar account was given by patient X, a dentist (F, 62 y.o.) who came in with suspicion of endoprosthesis allergy, which was not confirmed. Nevertheless, on general anamnesis, she reported an episode of acute eyelid dermatitis that lasted for more than a week after dyeing her eyebrows with “black henna” when she was 17 y.o. Further, two patients in group P3 were hairdressers with occupational allergies to hair dyes. One of them was presented above as patient R. The other one, patient Y, was a female hairdresser (18 y.o.) who complained of hand eczema that developed 2 years earlier, shortly after starting an apprenticeship in a hair salon. Soon after she started full-time employment, her dermatitis spread to her forearms, arms and face. In addition to the patch test results shown in Table 3, she also had positive reactions to five other hair dyes (Toluene–2,5–Diamine, 4–Amino–2–Hydroxytoluene, 4–Nitro–O–Phenylenediamine, 4–Aminophenol, 4–Amino–2–Hydroxytoluene), as well as Ammonium Persulfate—all of which were frequent occurrences in hair dye products available at that time in Poland [13]. Semi-open tests revealed contact dermatitis to three hair dye products, two shampoos and a hair-smoothing fluid from her salon. The last, somewhat different, case was patient Z, a laboratory medicine student (F, 20 y.o.), who was first exposed to paraphenylenediamine dihydrochloride during laboratory classes. A few months later, she experienced an episode of acute dermatitis on the scalp, ears, neck and hands after dyeing hair for the first time in her life. None of the seven patients manifesting a pattern of patch test reactivity described as P3 had any symptoms indicative of textile dermatitis.

In the pattern group P1, the percentage of clinically relevant patch test reactions to DBM amounted to 75.0%; there were no reactions to TDM 6.6%. In group P2, the figure was 66.7% for both DBM and TDM 6.6%. All patients in group P3 reacted to TDM 6.6%, but in none of them was it considered relevant; thus, the percentage of relevant reactions amounted to 0%. The remaining patient P in Table 3 did not fit any of the previous patterns: She had a positive reaction to TDM 6.6% but not to DBM or any other dyes listed in Table 3. Nevertheless, she was ultimately diagnosed with textile dermatitis based on clinical features (Figure 16). She might have been sensitised to a dye in her dark blue surgical gowns that was a component of TDM 6.6%. Unfortunately, she did not show up for further tests with individual components of TDM 6.6% and other textile dyes. As a single case, she was considered an exception, rather than a “pattern”.

### 3.5. Co-Positivity of Textile Dye Mix 6.6%

All 13 patients with positive tests with TDM 6.6% were also tested with DBM, and positive reactions to the latter were found in five (38.5%). When analysing the frequency of positive reactions to TDM 6.6% in patients who were positive to any of its components or the cross-reacting dyes, three textile dyes (DO3, DB106 and DO124) and three non-textile azo dyes passed the criterion of at least 10 patients with a positive patch test reaction. The co-positivity rates to TDM 6.6% were substantially higher among patients positive to DO3 and cross-reacting azo dyes, as compared to DB106, DB124 or their mix, DBM (Table 4).

When looking at cross-reactivity of DO3, the highest rate was observed among patients positive to the hair dye TDS, followed by 4AAB and PPD. In the case of disperse blue dyes, the co-positivity rates were all below 15% (Table 5).

In the final step of the analysis, co-positivity rates to DBM were checked among patients with positive patch tests to the individual components of the mix tested at higher concentrations (1% versus 0.5% in the mix). The results are shown in Table 6.

## 4. Discussion

The retrospective study design and mixed referral population in this article do not allow for conclusions regarding the prevalence of positive patch test results to textile dye tests among patients with dermatitis. Therefore, the primary focus was on clinical relevance, cross-reactivity, and the possible associations between them. The patch test positivity rates to TDM 6.6% reported in previous studies typically ranged from 0.5% to 18.2% [4,8,9,14,15,16]. Therefore, the positivity rate of 14.4% in the current analysis appears to be at the upper end of the range. This may be explained by selective referrals from other centres, as the author was one of the few dermatologists in Poland continuously using the textile series, as well as the hairdresser series, which included several cross-reactive hair dyes. The highest rates of positive reactions to TDM 6.6% were reported in a recent study of 716 Chinese children [17]. In this report, positivity rate to TDM 6.6% ranked first and to DBM fifth, with a significant male predominance observed in both: 38.1% males versus 29.6% females for TDM 6.6% and, respectively, 25.1% versus 17.4% for DBM [17]. Interestingly, the authors described a significant drop in the positivity rates with age, from 33.3% among children below 3 y.o., down to 17.6% among 12–14 y.o. A similar decreasing trend in positivity rates with age was observed among European children, but for metals rather than textile dyes [18]. The authors suggested a “loss of sensitisation” as a possible explanation for this decrease, but another likely explanation may be a decrease in false-positive reactions due to decreased susceptibility to irritant effects of metals in older children. The risk of false-positive reactions to textile dyes will be discussed below. Nevertheless, the positivity rates to TDM 6.6% among European children were lower by an order of magnitude (2.4–3.0%) than those reported in Chinese children; they were also relatively stable between age groups [18]. Differences between positivity rates reported in various studies may be due to genetic diversity and differences in exposure patterns, as well as other co-factors such as temperature, humidity and local dress code. They may also stem from varied inclusion criteria and discrepancies in reading and interpreting patch test reactions, although ICDRG criteria were cited in all studies. Focusing on clinical relevance rather than just positivity could arguably provide a more robust tool for investigating textile dye allergy. Patch test positivity seems an attractive outcome to study thanks to its relative simplicity and the widely accepted ICDRG criteria of positivity. In contrast to this, clinical relevance is a more elusive concept and depends on the knowledge, experience and inquisitiveness of the clinician, as well as the patient’s cognitive abilities and willingness to cooperate. On the other hand, only positive patch tests with current relevance, or those indicating relevant sensitisers due to cross-reactivity, can positively impact the patient’s quality of life.

The author is aware of only four studies of consecutive patients tested with TDM that reported on the clinical relevance of positive reactions. These were large, predominantly multi-centre studies with positivity rates ranging from 2.3 to 4.3% and relevant positive tests seen in 0.5–1.1% of patients, meaning that 12.5–31.5% of positive reactions were assessed as relevant to the patients’ current dermatitis [4,8,15,19]. These figures were higher among 209 patients with suspected textile dermatitis and amounted to 13.9% (positivity), 8.6% (relevance) and 61.9% (percentage of relevance among positive) [16]. The corresponding values in the current analysis are 14.4%, 4.4%, and 30.0%, which seem to better match the results of the latter study. Indeed, patients with suspected textile dermatitis were over-represented in the patient series due to referrals for this specific diagnostic procedure from other centres. As for DBM, positive reactions were found in 17.4% of patients, including relevant reactions in 10.6%; thus, 61.1% of positive reactions were clinically relevant.

**Figure 20:**
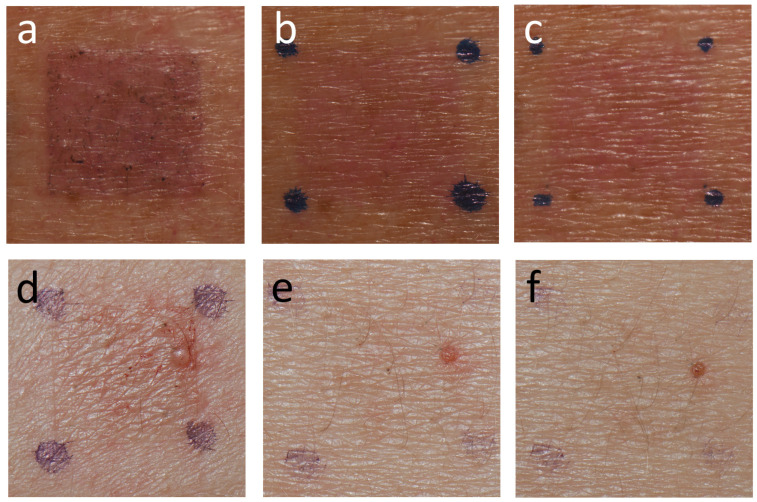
The pattern of reaction to DBM compared with a typical irritant reaction. The macular erythema-type reaction to DBM persisted from D3 (**a**) and D5 (**b**) through D8 (**c**). An irritant reaction to copper oxide 1% pet. healed rapidly from D3 (**d**) to D4 (**e**) and virtually disappeared on D5 (**f**).

In the present analysis, doubtful reactions described as macular erythema (ICDRG: “?+” or “?”) were regarded as negative, although some authors hold the opposite opinion. According to the author’s personal observations, DB106 and DB124, as well as DBM, frequently evoke skin reactions that can be defined as non-palpable erythematous macules, consistent with the description of a doubtful reaction on the ICDRG scale, which typically is considered as “not positive” [10]. Therefore, no further assessment of the clinical relevance of such reactions was undertaken for such reactions. On the other hand, some researchers suggest that macular erythema may actually be a manifestation of a relevant allergic reaction [20]. In a recent publication from the North American Contact Dermatitis Group (NACDG), nearly a half of doubtful reactions were ultimately interpreted as “allergic/positive” [21]. On the other hand, the reported rates of significance ascribed to such reactions vary between studies from 3.3% to 78.8% [22,23]. The prevailing argument for disregarding the doubtful reactions in the present analyses was the high number of macular, erythema-type reactions to disperse blue dyes, which seemed to support the assumption that they would be non-specific. As reported above, macular erythematous reactions were seen in 44.4% of patients patch-tested with TDM 6.6%, 31.9% with DBM, 13.0% with DB124, and 12.8% with DB106. On the other hand, the macular erythema-type reactions to disperse blue dyes oftentimes showed a tendency to persist, which is not consistent with the dynamics of irritant reactions, which typically follow the “decrescendo” (fading) pattern and heal rapidly after removal of the patches. In the case of textile dyes, it was somewhat striking that in many patients, the reaction persisted over the entire 5 days’ observation period from D3 to D8, as shown in Figure 20.

A possible explanation for such a persistent reaction to disperse blue dyes might be a putative vasoactive effect, sustained by slowly eluting deposits of the dye in the skin. Although there is no information on vasoactive effects of the disperse blue dyes in focus of this article, other azo dyes and their derivatives are known for a multitude of biological effects [24]. In an analysis of biopsies from 10 purpuric patch test reactions to DB124, DB106 or DB85, dilated blood vessels were found without signs of vasculitis in all skin samples [25]. However, the biopsies were taken from positive, rather than doubtful reactions, and the dilated capillaries were reportedly surrounded by an inflammatory infiltrate. It is not clear, therefore, whether this vasodilation was due to the biological activity of disperse blue dyes themselves or a part of an allergic inflammatory response. Another, more simplistic explanation may be that the bluish discolouration of the skin caused by the dyes might mimic a livedo response. Both these phenomena might also occur simultaneously. Regardless of the actual mechanisms involved, this persistent erythema-type pattern may pose a challenge even for an experienced patch tester.

**Figure 21:**
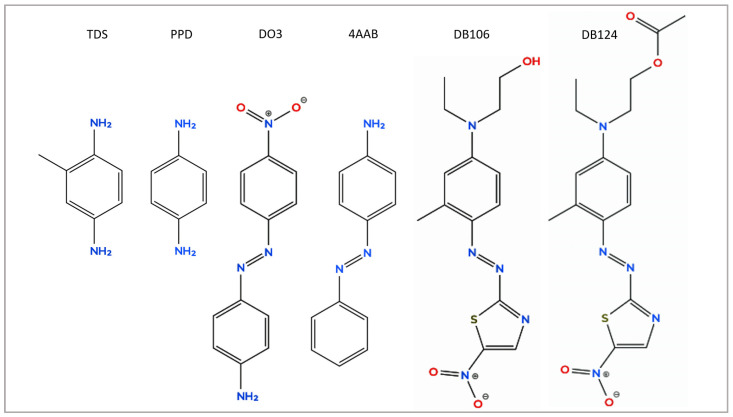
Molecular structures of azo dyes in focus of the present article.

The composition of TDM 6.6% introduced into the European Baseline Series in 2019 stemmed from more than two decades of multicentre research involving more than forty thousand patients [5]. Nevertheless, it has been debated since its introduction, especially with regard to possible cross-reactions between DO3 and other azo dyes, most notably the hair dyes PPD and TDS. As shown in Table 2, there were only weak positive reactions to TDM 6.6% among patients with confirmed textile dermatitis. The strongest reactions to TDM 6.6% were seen in patients Q and R, both diagnosed with a hair dye allergy rather than textile dermatitis. Table 4 shows that the rates of positive reactions to TDM 6.6% were substantially higher among patients with positive reactions to DO3 and cross-reacting azo dyes than to any other textile dye analysed. The cross-reactivity rate between the non-textile azo dyes (TDS, 4AAB, PPD) and TDM 6.6% was 3-fold higher than in the case of the disperse blue dyes (Table 5). This can be explained by the structural similarity of these dyes, with DB106 and DB124 appearing to be the most different from the others in the group (Figure 21).

Both TDS and PPD were frequent ingredients in hair dyes available in the period of this study. An analysis of 100 hair dyes of all shades (blond to black) sold in Poland in 2013 revealed that TDS was present in 55% and PPD in 29% of the products [13]. In the analysed period, 4AAB was prohibited in cosmetics in the EU; therefore, the observed positive patch tests were probably cross-reactions. Overall, cross-reactivity with non-textile (hair) dyes seemed to be a source of significant bias. In the analysed group, this bias was observed in 7 out of 13 TDM 6.6%-positive patients who demonstrated the reaction pattern described as P3 in Table 3. Common for these patients was a positive reaction to DO3, most probably due to cross-reactivity with TDS and PPD. In this group, there were two patients who seemed to have contracted their PPD allergy from temporary “henna” tattoos, notorious for being illegally laced with PPD [26]. Despite positivity to TDM 6.6%, none of these patients reacted to DBM, and none were ultimately diagnosed with textile dermatitis. In contrast to this, all 10 patients in groups P1 and P2 were DBM-positive, which coincided with the diagnosis of textile dermatitis in most of them. When looking at the eight patients in Table 3 with the ultimate diagnosis of textile dermatitis, positive reactions to DBM were seen in seven, of whom three did not react to TDM 6.6%. The only exception was patient P, who had a positive reaction to TDM 6.6% but not to DBM and whose ultimate diagnosis was textile dermatitis. To summarise Table 3, of the eight patients ultimately diagnosed with textile dye dermatitis, three cases would have been missed if screening had been performed using TDM alone (6.6%), compared with one case missed if screening had been performed using DBM alone. This observation speaks in favour of using DBM, rather than TDM 6.6%, as a screening marker of textile dye allergy. This may also explain why DBM, rather than TDM, is still included in important baseline series, including the British Standard Series, Chinese Baseline Series, Latin American Baseline Series and American Core Series.

On the other hand, it seems that DBM in the present formulation may also miss relevant cases of textile dermatitis. The mix contains DB106 and DB124 in lower concentrations (0.5% each) than their separate test preparations (each 1%). Table 1 shows that of the 13 patients with significant patch test reactions to DBM or any of its components, three patients (K, L, and M) would have been missed if only the mix had been used in patch testing. This is also illustrated in Table 6, where only 68.7% DB106-positive patients and 85.7% DB124-positive patients had concomitant positive reactions to DBM. TDM 6.6% contains DB106 and DB124 at even lower concentrations, 0.3% each, which seems to add to the problem. This is seen in patient F, who developed positive reactions to DB106 1% and DB124 1%, as well as to DBM (each 0.5%), but not to TDM 6.6%, which contained these dyes at 0.3% each (Table 2). This case of a confirmed textile dye allergy would have been missed if only tested with TDM 6.6%. Needless to say, both DBM and TDM 6.6% would miss patients with allergies to textile dyes other than their components. This was the case of patient S, in whom among all dyes in the contemporary textile series, sensitisation was found only to Acid Red 118. Other dyes from beyond the series might have also been involved as he reacted to fabrics in colours other than red (compare Figure 17).

When discussing possible limitations of the present study, its retrospective design has to be mentioned in the first place, along with a mixed population of consecutive patients with those who were referred for testing with suspected textile dermatitis. The observation period of 17 years means that batches of test haptens were replaced several times due to expiry dates. Some patients did not return for follow-up; therefore, the verdict on clinical relevance could not be verified in long-term observation. On the other hand, all tests were carried out by one experienced patch tester, who did all the tests personally—from qualifying patients for testing to loading haptens onto test chambers and placing these on the patient. The author performed all patch test readings on every observation point following the same ESCD protocol, as well as the assessments of clinical relevance of test results and, whenever possible, also reassessments during follow-up visits. Each patient and each positive patch test has been routinely photographed, which allowed for a retrospective verification of all previous readings and clinical patterns. Therefore, inter-observer variability seen in many studies was not an issue in the present study.

One of the proposed changes stemming from the present research, i.e., the removal of DO3 from the textile dye mix, has already been introduced in 2025. A second change, i.e., the increase of DB106 and DB124 concentrations in the mix up to 1% each, has recently been agreed upon by the European Baseline Series Working Group of the ESCD, and a respective recommendation has been submitted for publication in March 2026. Recent studies of patients tested in parallel with TDM 6.6%, TDM 5.6% and TDM 7% demonstrate that removal of DO3 from the mix results in a decrease in positivity rates, probably due to exclusion of cases cross-reactive with non-textile azo dyes, while the increased concentrations of DB106 and DB124 increase numbers of positive reactions, probably due to reduction in false negatives [8,9,19]. The final proposal arising from the present study is to double the concentrations of DB106 and DB124 from 0.5% to 1% in DBM. A possible drawback of such a change in both DBM and TDM may be an increased difficulty in reading patch tests and an increased number of questionable readings due to the persistent macular erythema-like reactions discussed above (Figure 20). Nevertheless, the increase of DB106 and DB124 concentrations to each 1% in the future TDM recommended by ESCD experts was founded on studies of 6 682 patients tested with TDM 7%, in which no increased frequency of irritant or false-positive reactions was observed [8,9,19]. By analogy, the same can be expected in the case of increasing the concentrations in DBM. Moreover, the chance of reducing numbers of patients with textile dye allergies who are missed by the present screening mixes would offset a possible inconvenience of reading, which can be compensated by a thorough assessment of clinical relevance. The proposed changes are summarised in Table 7.

After collecting and analysing the presented data, the manufacturer of the patch test materials used in this study (Chemotechnique Diagnostics) announced a change in the labelling with regard to concentrations of textile dyes. According to the producers’ information, the change was a correction of calculations without actual change in the concentration of haptens in the test material. Products with old and new labels are declared as fully interchangeable, and their catalogue numbers remain unchanged. Up to this point, the author has adhered to the concentration descriptions consistent with those declared on the labels in the period covered by this retrospective analysis (2007–2024). However, to place the conclusions in the current context, the proposed changes are translated into “new” concentrations in Table 8, and the final conclusions will also be expressed in accordance with the new labelling of 2026.

## 5. Conclusions

Contact dermatitis caused by textile dye allergy appears to be underdiagnosed. This problem can be addressed by raising awareness of this condition among healthcare professionals and by improving screening tools for textile dye allergy used in consecutive patients as part of routine patch testing. Based on the results of this study, proposed improvements include the following:

- Removing Disperse Orange 3 from the textile dye mix, which was done in the meantime;
- Tripling the concentrations of Disperse Blue 106 and Disperse Blue 124 in the textile dye mix;
- Doubling the concentrations of Disperse Blue 106 and Disperse Blue 124 in the Disperse Blue Mix 106/124.

## Figures and Tables

**Table 1:** Positive reactions to Disperse Blue Mix (DB106 and DB124, each 0.5% pet.) or its components DB106 and DB 124 (each 1% pet.).

Dyes Tested	Reaction Patterns						
DBM 106/124 1%	+	+	+	?	?	–	+
DB106 1%	+	?	+	+	+	+	–
DB124 1%	+	+	–	+	?	–	–
No. of patients	10	2	1	1	2	1	2
No. of patients with relevant reactions ^1^	7	2	1	1	1	1	0
Patients ^2^ with relevant results	A–G	H, I	J	K	L	M	–

^1^ A positive patch test was considered clinically relevant (CODEX: C) when the patient was ultimately diagnosed with textile dermatitis and the dye was deemed a probable cause for it. ^2^ The capital letters symbolise patients whose descriptions are provided further in this article.

**Table 2:** Patch test results in 10 patients tested with TDM 6.6% and all its components, as well as three azo dyes cross-reactive with Disperse Orange 3.

Haptens	Patients									
	D	G	F	Q	R	S	–	–	–	–
TDM 6.6%	+	+	–	++	+++	?	?	?	–	–
DB35 1%	–	–	–	–	–	–	–	–	?	–
DO1 1%	–	–	–	–	–	–	–	–	–	–
DO3 1%	–	–	–	+	+++	–	–	–	–	?
DR1 1%	–	–	–	–	–	–	–	–	–	–
DR17 1%	–	–	–	–	?	?	–	–	–	?
DY3 1%	–	–	–	–	–	–	–	–	–	–
DB106 1%	+	+	+	–	–	–	–	?	–	–
DB124 1%	+	+	+	–	–	–	–	?	–	–
DBM 1%	+	+	+	?	–	–	?	?	–	–
4AAB 0.25%	?	–	–	+++	+++	–	–	–	–	–
PPD 1%	–	–	–	+	+++	–	–	–	–	–
TDS 1%	–	–	–	+	+++	–	–	–	–	–
Sex	M	F	M	M	M	M	F	F	M	F
Age (y.o.)	53	27	15	27	34	12	16	41	29	27
Textile dermatitis?	Yes	Yes	Yes	No	No	Yes	No	No	No	No
Relevance ^1^	C	C	C	X	X	–	–	–	–	–

^1^ Clinical relevance: C, current relevance; X, cross-reaction.

**Table 3:** DBM, TDM 6.6% versus DO3 and cross-reactive azo dyes.

Haptens	Patients																	
	F	T	U	–	D	G	M	O	–	–	Q	R	V	W	X	Y	Z	P
DBM 1%	+	+	+	+	+	+	+	+	+	+	?	–	?	?	?	–	–	?
TDM 6.6%	–	?	?	?	+	+	+	+	+	+	++	+++	+	+	+	+	++	+
DO3 1%	–	–	?	–	–	–	–	–	–	–	+	+++	+	+	+	+	+++	–
PPD 1%	–	–	–	–	–	–	–	–	–	–	+	+++	+	+	+	++	+++	–
4AAB 0.25%	–	–	–	–	?	–	–	–	–	–	++	+++	+	+	+	+	+++	–
TDS 1%	–	–	–	–	–	–	–	–	–	–	+	+++	+	+	+	+	++	–
Sex	M	F	M	F	M	F	F	F	F	F	M	M	F	F	F	F	F	F
Age (y.o.)	15	33	14	44	53	27	25	11	16	36	27	34	33	36	61	18	21	30
Textile dermatitis?	Yes	Yes	Yes	No	Yes	Yes	Yes	Yes	No	No	No	No	No	No	No	No	No	Yes
Relevance ^1^	C	C	C	D	C	C	C	C	D	D	X	X	X	X	X	X	X	C
Group patterns ^2^	P1				P2						P3							–

^1^ Clinical relevance: C, current relevance; D, doubtful (unknown); X, cross-reaction. ^2^ Patterns of patch test reactions are discussed in the text below.

**Table 4:** The frequency of positive reactions to TDM 6.6% among patients positive to its individual components or azo dyes cross-reacting with DO3.

Dyes Tested	Total (+) ^1^	F	M	TDM 6.6% (+)	F ^2^	M ^2^
4AAB 0.25%	12	8	4	7 (58.3%)	5 (62.5%)	2 (50.0%)
DO3 1%	13	9	4	7 (53.8%)	5 (55.6%)	2 (50.0%)
TDS 1%	14	9	5	7 (50.0%)	5 (55.6%)	2 (40.0%)
PPD 1%	21	16	5	7 (33.3%)	5 (31.2%)	2 (40.0%)
DBM 1%	36	24	12	6 (16.7%)	5 (20.8%)	1 (8.3%)
DB124 1%	14	7	7	2 (14.3%)	1 (14.3%)	1 (14.3%)
DB106 1%	16	8	8	2 (12.5%)	1 (12.5%)	1 (12.5%)

F, females; M, males. ^1^ Results are shown only for subgroups of no less than 10 patients positive to a given dye. ^2^ Percentages of positive patch test results calculated within the gender subgroups.

**Table 5:** The frequency of positive reactions to DO3 among patients positive to individual components TDM 6.6% and cross-reactive azo dyes.

Dyes Tested	Total (+) ^1^	F	M	DO3 (+)	F ^2^	M ^2^
TDS 1%	14	9	5	11 (78.6%)	7 (77.8%)	4 (80.0%)
4AAB 0.25%	12	8	4	9 (75.0%)	6 (75.0%)	3 (75.0%)
PPD 1%	21	16	5	12 (57.1%)	8 (50.0%)	4 (80.0%)
DB124 1%	14	7	7	2 (14.3%)	2 (28.6%)	0
DB106 1%	16	8	8	2 (12.5%)	2 (25.0%)	0
DBM 1%	36	24	12	4 (11.1%)	3 (12.5%)	1 (6.7%)

F, females; M, males. ^1^ Results are shown only for subgroups of no less than 10 patients positive to a given dye. ^2^ Percentages of positive patch test results calculated within the gender subgroups.

**Table 6:** The frequency of positive patch test reactions to DBM 1% pet. (DB106 and DB124, each 0.5%), among patients with positive reactions to DB106 1% pet. and DB124 1% pet.

Dyes Tested	Total (+) ^1^	F	M	DBM (+)	F ^2^	M ^2^
DB124 1%	14	7	7	12 (85.7%)	6 (85.7%)	6 (85.7%)
DB106 1%	16	8	8	11 (68.7%)	6 (75.0%)	5 (62.5%)

F, females; M, males. ^1^ Results are shown only for subgroups of no less than 10 patients positive to a given dye. ^2^ Percentages of positive patch test results calculated within the gender subgroups.

**Table 7:** The different compositions of textile dye mix and disperse blue mix used in this study (TDM 6.6% and DBM 1%), the updated TDM introduced in 2025 (TDM 5.6%) and proposed compositions based on the results from the present study (TDM 7% and DBM 2%) and other studies (TDM 7%); concentrations of textile dyes are expressed according to labels in force during the study period.

Composition	TDM 6.6%	TDM 5.6%	TDM 7%	DBM 1%	DBM 2%
CT product number	Mx–30	Mx–32	–	Mx–26	–
Period of usage	2019–2024	2025 –present	proposed	2007 –present	proposed
DB35	1%	1%	1%	–	–
DO1	1%	1%	1%	–	–
DO3	1%	–	–	–	–
DR1	1%	1%	1%	–	–
DR17	1%	1%	1%	–	–
DY3	1%	1%	1%	–	–
DB106	0.3%	0.3%	1%	0.5%	1%
DB124	0.3%	0.3%	1%	0.5%	1%

**Table 8:** The different compositions of textile dye mix and disperse blue mix used in this study, the updated textile dye mix introduced in 2025 and the proposed compositions based on the results from the present study and other studies. The concentrations of textile dyes are expressed in accordance with product labels of 2026.

Composition	TDM 1.75% (ex 6.6%)	TDM 1.6% (ex 5.6%)	TDM 1.95%	DBM 0.25%	DBM 0.5%
CT product number	Mx–30	Mx–32	–	Mx–26	–
Period of usage	2019–2024	2025 –present	proposed	2007 –present	proposed
DB35	0.1%	0.1%	0.1%	–	–
DO1	0.15%	0.15%	0.15%	–	–
DO3	0.15%	–	–	–	–
DR1	0.4%	0.4%	0.4%	–	–
DR17	0.4%	0.4%	0.4%	–	–
DY3	0.4%	0.4%	0.4%	–	–
DB106	0.075%	0.075%	0.25%	0.125%	0.25%
DB124	0.075%	0.075%	0.25%	0.125%	0.25%

## Data Availability

The data presented in this study are available on request from the corresponding author due to medical confidentiality and the General Data Protection Regulation (EU 2016/679).

## Abbreviations

The following abbreviations are used in this manuscript:

4AAB	4–Aminoazobenzene
CODEX	Grading system of a positive patch test’s clinical relevance
DB35	Disperse Blue 35
DB85	Disperse Blue 85
DB106	Disperse Blue 106
DB124	Disperse Blue 124
DBM	Disperse Blue Mix (106/124)
DO1	Disperse Orange 1
DO3	Disperse Orange 3
DO34	Direct Orange 34
DR1	Disperse Red 1
DR17	Disperse Red 17
DY3	Disperse Yellow 3
ESCD	European Society of Contact Dermatitis
ICDRG	International Contact Dermatitis Research Group
PPD	para–Phenylenediamine
PT	patch test, patch testing
TDM 5.6%	Textile Dye Mix 5.6%
TDM 6.6%	Textile Dye Mix 6.6%
TDM 7%	Textile Dye Mix 7%
TDS	Toluene 2,5–Diamine Sulfate
